# Beta-Thalassemia in Adulthood Previously Suspected as Treatment-Resistant Iron Deficiency Anemia: A Case Report

**DOI:** 10.7759/cureus.60275

**Published:** 2024-05-14

**Authors:** Hitesh P Rai, Anthony J Stuart

**Affiliations:** 1 Research, Edward Via College of Osteopathic Medicine, Monroe, USA; 2 Internal Medicine, Willis Knighton Tri-State Medical Clinic, Shreveport, USA

**Keywords:** chronic conditions, beta thalassemia, hematology, primary care, internal medicine

## Abstract

Beta-thalassemia (β-thalassemia) is a hematologic genetic condition that causes microcytic anemia due to defective synthesis of the hemoglobin beta chain. As a hypochromic microcytic anemia that is commonly associated with symptoms such as fatigue and pallor when identified in adulthood, β-thalassemia may be commonly underdiagnosed or misdiagnosed as iron deficiency anemia. This study presents a case of a patient with β-thalassemia who was initially misdiagnosed with treatment-resistant iron deficiency anemia.

Here, we present the case of a 66-year-old male of Mediterranean descent with a history of military service who presented with persistent fatigue. He had a past medical history of hypertension, diabetes mellitus type 2, sleep apnea, and iron deficiency anemia. Despite undergoing unnecessarily prolonged iron supplementation for suspected iron deficiency anemia, the patient’s complete blood count and peripheral blood smear continued to identify hypochromic microcytic anemia. Ultimately, hemoglobin electrophoresis was performed, and mutations were identified in the hemoglobin beta chain consistent with β-thalassemia minor.

Due to its rarity and wide variation in presentation, β-thalassemia may be frequently misdiagnosed. β-thalassemia is a spectrum of disorders ranging from β-thalassemia minor, which may be asymptomatic and incidentally discovered in adulthood, to β-thalassemia major, which may include bone marrow deformities from extramedullary hematopoiesis and require frequent blood transfusions to sustain life. Therefore, patients who present with symptoms of β-thalassemia minor may not be identified until later in life after undergoing decades of ineffective treatment.

β-thalassemia is a multifactorial disease with a variety of clinical presentations that can easily be misdiagnosed as other types of anemia. This case highlights the importance of performing thorough laboratory testing and casting a wide net of differential diagnoses when evaluating patients with treatment-resistant anemia. This case calls for further research on the genetic contributions to β-thalassemia as well as improved ways to identify this disorder, particularly in patients who may not have a severe form that is easily diagnosed in early childhood.

## Introduction

Beta-thalassemia (β-thalassemia) is a categorical classification of heterogeneous autosomal recessive hereditary anemias present due to reduced or absent synthesis of the β-globin chain in hemoglobin [1]. The three main presentations of β-thalassemia include the following: β-thalassemia major (TM), also commonly known as “Cooley’s anemia” or “Mediterranean anemia”; β-thalassemia intermedia (TI); and β-thalassemia minor (TMi), also referred to as “β-thalassemia carrier” or “β-thalassemia trait” [1]. Overall, the prevalence of β-thalassemia is quite rare; a mere 1.5% of the global population carries the β-thalassemia trait, with approximately 60,000 symptomatic individuals born annually [2]. The clinical presentation of patients with β-thalassemia varies tremendously, based on both individual factors and gene homozygosity [3].

Individuals born with TM usually present before two years of age with severe anemia that requires regular red blood cell (RBC) transfusions to avoid complications such as growth retardation, jaundice, hypotonia, hepatosplenomegaly, ulceration of the lower extremities, masses secondary to extramedullary hematopoiesis, skeletal alterations resulting from bone marrow expansion, and heart failure [1,4].

Individuals with TI are characterized with a clinical polymorphism, attributable to the condition’s heterogeneity [5]. These patients may experience ineffective erythropoiesis, chronic anemia, iron overload present with splenomegaly, extramedullary erythropoiesis, and thrombophilia, and require a tailored approach to therapy, given their individual presentation and age [5,6].

Individuals with TMi are often asymptomatic or may have mild symptoms of anemia without significant physical exam findings [7]. TMi is usually discovered incidentally on routine complete blood count [7]. As a result of its mild or otherwise absent presentation, TMi presents similarly as iron deficiency anemia and may be treated as such; therefore, a distinction between the two conditions is crucial in reducing inappropriate iron therapy or unnecessary diagnostic procedures [8].

Diagnosis of β-thalassemia requires a comprehensive evaluation; failure to appropriately diagnose and treat this condition may lead to increased complications of the disease, such as exacerbation of other conditions including diabetes mellitus [9]. Here, we present the case of a 66-year-old male of Mediterranean descent with a history of military service who presented with persistent fatigue. He had a past medical history of hypertension, diabetes mellitus type 2, sleep apnea, and iron deficiency anemia. Through this case, we highlight the importance of early detection and treatment of β-thalassemia minor and the potential consequences of leaving this condition untreated.

## Case presentation

A 66-year-old male of Mediterranean descent presented to the primary care clinic to establish care and for the management of chronic conditions. The patient’s past medical history included type 2 diabetes mellitus without complications, mixed hyperlipidemia, essential hypertension, angina pectoris, gastroesophageal reflux disease without esophagitis, obstructive sleep apnea, depressive disorder, left knee pain, colonic polyps, anemia, and unspecified β-thalassemia. The patient was a military veteran and reported receiving care through the local Veterans Affairs hospital in the past. He reported having undergone previous treatment for anemia, which consisted of iron supplementation that did not correct his hemoglobin values.

Past surgical history included left knee surgery and tonsillectomy. Family history of medical conditions included colonic polyps, arthritis, and cardiovascular disease. The patient had no known history of anemia or bleeding disorders in his family. The patient was divorced and denied the use of tobacco, alcohol, or illicit drug products. The patient was up to date on all immunizations and age-appropriate screening tests.

At the patient’s initial appointment, he provided his past medical history and denied taking any medications besides a calcium supplement at that time. The patient had no known drug allergies, and a review of the systems was negative across all different systems.

On physical examination, the patient was well-developed and well-nourished, with no acute distress. His vitals revealed a blood pressure of 192/98 mmHg, pulse of 86 bpm, temperature of 98.6 F, and O_2_ saturation of 97% on room air. The patient weighed 225.6 lbs and was 5’11”, with a body mass index (BMI) of 31.46.

Initial assessment of the patient included type 2 diabetes mellitus, mixed hyperlipidemia, and essential hypertension, all of which were unmanaged at the time. Medication management was provided for these conditions including metformin, losartan-hydrochlorothiazide, nebivolol, amlodipine, and rosuvastatin. Additionally, laboratory services ordered included hemoglobin A1c, complete blood count, acute hepatitis panel, comprehensive metabolic panel, liver function tests, direct bilirubin, total bilirubin, lipid panel, prostate serum antigen, thyroid stimulating hormone, urinalysis with reflex microscopy and culture, and random urine microalbumin. A hemoglobin electrophoresis was ordered as well, given the patient’s history. The patient was also referred to gastroenterology for an esophagogastroduodenoscopy (EGD).

The patient’s lab values were significant for direct hyperbilirubinemia, and an elevated hemoglobin A1c. Additionally, a summary of his complete blood count is provided below in Table 1. The patient’s lipid panel provided values within normal limits and blood pressure had decreased to 148/80 mmHg from 192/98 mmHg at the initial visit. The patient’s EGD revealed esophagitis and gastritis. Pioglitazone was added to the patient’s medication list and his dose of nebivolol was increased to provide better control of the patient’s diabetes mellitus and hypertension. 

**Table 1:** Complete blood count

Unit	Value
White blood cells	6.0*10^3^ /uL
Red blood cells	5.41*10^6^ /uL
Hemoglobin	11.4 g/dL
Hematocrit	35.2%
Mean corpuscular volume	65.0 fL
Mean corpuscular hemoglobin	21.1 pg
Mean corpuscular hemoglobin concentration	32.5 g/dL
Red cell distribution width	17.5%
Platelet count	256*10^3^ /uL
Mean platelet volume	9.0 fL
Neutrophil %	58.9%
Lymphocyte %	29.6%
Monocyte %	7.9%
Eosinophil %	2.5%
Basophil %	1.1%

**Figure 1:**
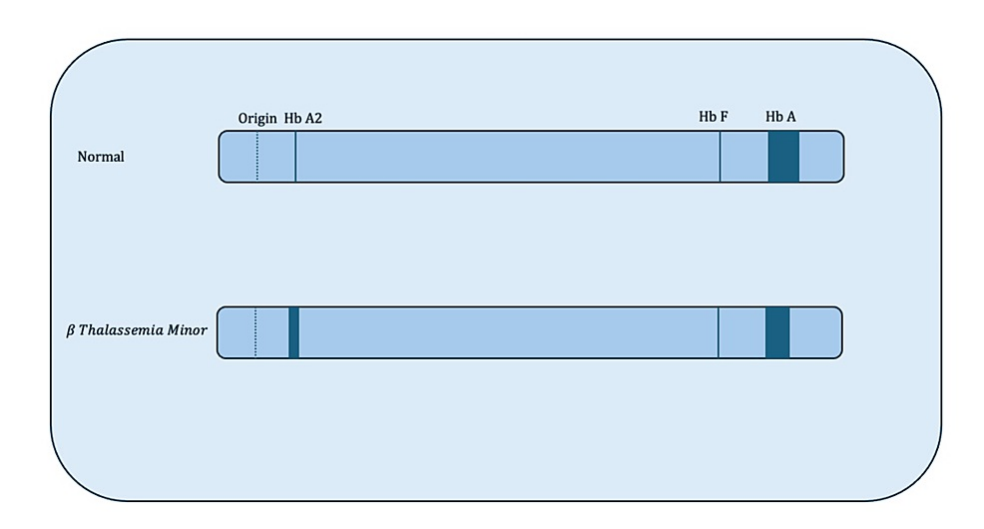
Depiction of hemoglobin electrophoresis results consistent with beta-thalassemia minor HbA2: Hemoglobin A2; HbA: Hemoglobin A; HbF: Hemoglobin F

Hemoglobin electrophoresis revealed β-thalassemia minor. Figure 1 depicts hemoglobin electrophoresis consistent with β-thalassemia minor, and the specific hemoglobin values are listed in Table 2. Thus, management of the patient's β-thalassemia minor consists of observation and monitoring of his elevated liver enzymes. If he continues to stay otherwise asymptomatic, his prognosis regarding his β-thalassemia minor would be good.

**Table 2:** Hemoglobin electrophoresis test values

Hemoglobin	Value
Hemoglobin A	93.3%
Hemoglobin A2	5.1%
Hemoglobin F	1.6%

Repeat laboratory tests were ordered, including urinalysis with reflex microscopy, hemoglobin A1c, comprehensive metabolic panel with liver function tests, complete blood count, and lipid panel. The patient was instructed to continue management of his chronic conditions with the medications prescribed and return for a follow-up appointment in three months. The patient provided written consent for his information to be used in the report of this case.

## Discussion

This patient’s hematological condition, although rare, would otherwise be overlooked. He reported that it was initially treated as iron deficiency anemia at the local Veterans Affairs hospital before being diagnosed again as β-thalassemia minor. There is tremendous overlap of symptoms between hypochromic microcytic anemias, and it is important to have a wide differential diagnosis, especially when addressing anemias resistant to standard treatments. The Mentzer Index may be utilized to distinguish between iron deficiency anemia and β-thalassemia; it compares the mean corpuscular volume to the RBC count, and a value greater than 13 suggests iron deficiency anemia, whereas a value below 13 suggests β-thalassemia [10]. In cases where there is a co-occurrence of both iron deficiency anemia and β-thalassemia, additional testing such as hemoglobin electrophoresis and peripheral blood smear may be utilized [11]. In this patient's case, however, the value was 12, which is consistent with the given condition. What may appear at first as a minuscule abnormal laboratory finding could ultimately play a larger role in a patient’s overall health.

Patients with TM are homozygotes or compound heterozygotes for β0 or β+ genes, while patients with TI are mostly homozygotes or compound heterozygotes, and patients with TMi are mostly heterozygotes [1]. β-thalassemia historically has had a higher prevalence in particular regions of the world, including the Mediterranean, Middle East, and Southeast Asia; however, the prevalence is increasing in regions such as North America and Northern Europe, largely as a result of migration [2].

Diagnosis of β-thalassemia requires a comprehensive evaluation shaped by the presence of microcytic hypochromic anemia, distinction from iron deficiency, anisopoikilocytosis with nucleated RBCs on peripheral blood smear, and reduced or complete absence of hemoglobin A (HbA), increased hemoglobin A2 (HbA2), and often hemoglobin F (HbF) on hemoglobin analysis [11]. Molecular analysis may be utilized to determine globin genotype as well [7]. A detailed patient history and physical exam that include screening for pallor, poor weight gain, stunted growth, mild jaundice, and hepatosplenomegaly are critical prior to laboratory testing to aid in diagnosing β-thalassemia [11]. The differential diagnosis for β-thalassemia includes similarly presenting hematological conditions, including alpha-thalassemia (α-thalassemia), iron deficiency anemia, and sideroblastic anemias [1]. In this case, the patient had undergone routine laboratory testing, but a hemoglobin electrophoresis was not ordered to diagnose this condition until after prolonged treatment for perceived iron deficiency anemia. Tests such as hemoglobin electrophoresis should always be considered in the case of treatment-resistant anemia regardless of the patient’s age.

While this patient has a milder form of β-thalassemia that may be asymptomatic, it is likely that this patient’s hematological condition laid the groundwork for other disease states, such as diabetes mellitus, to develop. Current research suggests a correlation between diabetes mellitus and β-thalassemia minor as the risk of developing diabetes and insulin resistance in patients with β-thalassemia minor is two times greater than in the general population [9]. Inflammation caused by beta-thalassemia may occur in liver cells and leave patients susceptible to insulin resistance, impaired glucose tolerance, and ultimately diabetes [9].

Furthermore, the patient’s history of left knee pain may be another presenting manifestation of β-thalassemia; thalassemia bone disease may affect all aspects of bone health, including anatomy, quality, and density, which increase the risk for osteoporosis, fractures, spinal deformities, nerve compression, and pain [12].

Additionally, β-thalassemia minor may serve as a protective factor against other pathological conditions. A few studies suggest that β-thalassemia may provide some protection against cerebrovascular accidents via reduced total cholesterol and LDL levels alongside an association with decreased prevalence of hypertension in β-thalassemia minor patients [13]. Although not the case with this patient, as he has both hypertension and hyperlipidemia, there surely is a consideration for the protective factors β-thalassemia minor may provide. There is also a plethora of studies exploring the protective effects thalassemia disorders may provide against malarial infections. There is strong evidence that β-thalassemia traits increase resistance to *Plasmodium falciparum* infections, although the cellular mechanisms are not clear [14]. Although this patient is not in a malaria-endemic region, his case provides insight into the epidemiology and survival of the trait and its succession across generations.

Management and treatment of β-thalassemia varies by its severity in presentation [15]. For those with TM, treatment consists of routine blood transfusion, bone marrow transplantation, iron chelation therapy, hematopoietic stem cell transplantation, fetal hemoglobin production stimulation, and gene therapy [15]. The target of transfusion is primarily to suppress erythroid expansion, mitigate symptoms of anemia, and inhibit gastrointestinal iron absorption [7]. For those with TI, treatment is symptomatic; it can be accomplished via folic supplementation or splenectomy [15]. For those with TMi, management often focuses on genetic counseling and prenatal diagnosis if indicated when carriers are detected [8]. Furthermore, it is imperative to follow up with patients undergoing treatment for potential adverse events via monthly physical examinations, liver function testing every two months, serum ferritin testing every three months, and growth and development milestones every six months (in pediatric patients) [7]. In patients with β-thalassemia ages 10 and older, complete cardiac evaluation, thyroid, parathyroid, pancreatic, adrenal, and pituitary function evaluation, liver ultrasound, serum alpha-fetoprotein for early detection of hepatocarcinoma, and bone densitometry for osteoporosis are recommended to be performed annually [16]. Given the recommendations, it is important to treat and monitor patients with TMi so that other disease states can be prevented from early detection and treatment.

The prognosis of β-thalassemia has dramatically improved over recent decades due to the widespread availability of transfusion regimens and effective iron chelation therapy [17]. The prognosis continues to improve as access to treatment increases; however, life expectancy remains low in settings that lack resources, with more than half of individuals dying before age 30 compared to more than half of individuals living to age 60 in settings that have greater resources [18]. Individuals who do not receive RBC transfusions usually die in the first two decades of life [10]. While the prognosis for individuals with β-thalassemia has substantially improved over the last two decades in parallel with medical advances in transfusion, iron chelation, and bone marrow transplantation therapy, cardiac disease remains the main cause of death in those with iron overload [4].

Thus, it is important to consider the role β-thalassemia may play in this patient's chronic conditions. New insights into the correlation between diabetes and β-thalassemia may serve as a justification for additional screening in patients with β-thalassemia for diabetes and its associated pathological disease processes.

Future studies should consider focusing on uncovering a more accurate rate for β-thalassemia minor since it is underdiagnosed as well as testing for other conditions secondary to the anemia and associated inflammation. Perhaps studies with larger groups and interventional screening in randomized controlled trials can determine the efficacy of such screening in patients with β-thalassemia minor as well as determine whether genetic screening for β-thalassemia is justifiable in patients with unspecified microcytic hypochromic anemia. This case’s importance explains the rare yet benign hematological condition and the role it plays in other chronic conditions.

## Conclusions

Although this patient’s chronic medical conditions of diabetes and joint pain are not uncommon across patients with similar demographics, his underlying β-thalassemia minor and how it may play a role in the underlying physiology behind his conditions surely is. A “one size fits all” approach toward primary care delivery would be provincial; thus, it is important to keep in mind how an uncommon hematological condition may or may not modify decisions made regarding patient care. This patient’s particular case of β-thalassemia minor, alongside his other conditions, may benefit in reminding us of the bigger picture of patient health and all its pieces to promote providing optimal holistic primary care services to other patients with mild yet rare conditions.
